# Increased precision of orthotopic and metastatic breast cancer surgery guided by matrix metalloproteinase-activatable near-infrared fluorescence probes

**DOI:** 10.1038/srep14197

**Published:** 2015-09-23

**Authors:** Chongwei Chi, Qian Zhang, Yamin Mao, Deqiang Kou, Jingdan Qiu, Jinzuo Ye, Jiandong Wang, Zhongliang Wang, Yang Du, Jie Tian

**Affiliations:** 1 Key Laboratory of Molecular Imaging of Chinese Academy of Sciences, Institute of Automation, Chinese Academy of Sciences, Beijing 100190, China; 2 School of Life Science and Technology, Xidian University, Xi’an 710071, China; 3 Department of General Surgery, General Hospital of People’s Liberation Army, Beijing 100853, China

## Abstract

Advanced medical imaging technology has allowed the use of fluorescence molecular imaging-guided breast cancer surgery (FMI-guided BCS) to specifically label tumour cells and to precisely distinguish tumour margins from normal tissues intra-operatively, a major challenge in the medical field. Here, we developed a surgical navigation system for real-time FMI-guided BCS. Tumours derived from highly metastatic 4T1-luc breast cancer cells, which exhibit high expression of matrix metalloproteinase (MMP) and human epidermal growth factor receptor 2 (HER2), were established in nude mice; these mice were injected with smart MMP-targeting and “always-on” HER2-targeting near-infrared (NIR) fluorescent probes. The fluorescence signal was imaged to assess *in vivo* binding of the probes to the tumour and metastatic sites. Then, orthotopic and metastatic breast tumours were precisely removed under the guidance of our system. The post-operative survival rate of mice was improved by 50% with the new method. Hematoxylin and eosin staining and immunohistochemical staining for MMP2 and CD11b further confirmed the precision of tumour dissection. Our method facilitated the accurate detection and complete removal of breast cancer tumours and provided a method for defining the molecular classification of breast cancer during surgery, thereby improving prognoses and survival rates.

According to a report by the National Cancer Institute (NCI), breast cancer is the third most frequently diagnosed cancer, with an estimated 1.38 million new cases per year^1^. Although the Department of Defence and NCI provide substantial financial support for breast cancer research^2^, the mortality rate is still increasing in particular regions^3^. The primary treatment modality for most breast tumours is surgery. Evidence has suggested that insufficient detection of breast tumour margins, which include irregular and indistinct tumour margins and metastases, is associated with poor prognosis^4^. Traditional surgery leads to either incomplete dissection of tumours or unnecessary removal of healthy tissue. If the pathological examination confirms that the tumour margin is more than 1 mm from the resection edge, complete resection has been achieved. Otherwise, additional tissue resection should be performed. However, this intra-operative evaluation is time consuming, dependent on the surgeon’s experience, and unreliable compared with post-operative pathological analysis. Indeed, several studies have shown that about 20–50% of breast cancer patients have positive tumour margins after lumpectomy^5^,^6^,^7^,^8^. Therefore, the development of more accurate surgical methods could improve the detection and radical resection of orthotopic and especially metastatic breast tumours^9^. Although surgeons and researchers have recently begun to focus on new methods for quickly, accurately, and objectively locating breast tumour margins^10^,^11^, the ever-increasing interest in the cooperation between medical imaging and surgery supports the need to develop an effective and efficient surgical method that can accurately detect tumour margins with high sensitivity and selectivity during surgery^12^.

Existing medical imaging technology has played an important role in pre-operative diagnosis and post-operative assessment. However, few current medical imaging methods, such as radiography, computed tomography, magnetic resonance imaging, positron emission tomography, and single-photon emission computed tomography, have been designed for intra-operative use by surgeons due to the limitations of machine size or radiation in application of intra-operative image-guided surgery^13^,^14^. Molecular imaging (MI) has been developed as a powerful tool for surgeons to precisely detect breast tumour tissues^15^. With tumour-specific probes, (sub)cellular-level breast tumour specimens can be detected, dissected, and intra-operatively evaluated using image-guided approaches^16^. Recent studies have supported the use of fluorescence MI (FMI) during surgery to overcome the challenges associated with precise intra-operative detection of tumour margins^17^ due to the following advantages: (1) FMI is non-radioactive, non-invasive, highly sensitive, and specific for tumour detection, and many studies have used this technique for detection of different types of tumours^18^,^19^,^20^,^21^,^22^,^23^; (2) FMI is a simple, robust approach that is well suited for intra-operative imaging-guided surgery; and (3) compared with other imaging techniques, FMI-guided breast cancer surgery (BCS) can realise the detection of early lesions *in vivo* and has been shown to be advantageous for the intra-operative, accurate localisation of tumour margins and the precise resection of lesions^24^,^25^.

Tumour margin detection is dependent on precise prognosis of irregular orthotopic tumours and metastases during surgery^26^. Matrix metalloproteinases (MMPs) are known to play a critical role in tumour progression, invasiveness, and metastasis^27^ and are promising tumour biomarkers overexpressed in most cancers^28^. MMPs degrade collagen type IV and further enable the escape of cancer cells to other organs^29^. Additionally, MMP probes have been shown to decrease the incidence of positive margins and increase tumour-free survival after dissections. Therefore, MMP can be used as a specific targeting site for delineating the tumour margin.

To evaluate the function and efficacy of our FMI-guided BCS technique for the precise detection and complete resection of breast metastatic tumours, we used a smart fluorescent probe, MMPSense 750 FAST (hereafter referred to as MMP-750; PerkinElmer, Inc., Waltham, MA, USA) to target MMP based on the following three reasons: (1) the probe can efficiently and abundantly accumulate at tumour sites due to the enhanced permeability and retention effect (EPR); (2) MMP-750 is a MMP-activatable imaging agent that is optically silent upon injection and produces a fluorescent signal after cleavage by MMP enzyme (with a high signal-to-background ratio); and (3) near-infrared (NIR) fluorescence, emitted at 750 nm, eliminates autofluorescence from the body, which can increase the accuracy of imaging data collected from biomedical applications. Additionally, NIR is undetectable by human eyes, but visible on the display monitor, facilitating simple operation procedures and minimising the learning curve.

Distinguishing the subtype of breast cancer intra-operatively could provide immediate information to the surgeon and oncologist regarding further optimal therapeutic options. Therefore, the breast cancer-specific receptor human epidermal growth factor receptor 2 (HER2) was chosen as another targeting site for determining the molecular classification of breast cancer. Herein, an always-on probe, HER2Sense 645 (hereafter referred to as HER2-645; PerkinElmer, Inc.), was used to positively and specifically target HER2.

The 4T1 breast orthotopic and metastatic tumour model was used as a target model system. We found that the probes could abundantly accumulate in the tumour due to the EPR or positive targeting effects of the different probes, and the probes would stably reside in the tumour for a long time. This allowed localisation of the whole breast tumour for pre- and intra-operative visualisation with FMI and the discrimination between malignant and normal tissue types. Moreover, immediate recognition of the subtype of breast cancer could be achieved intra-operatively, and excised tissues could be further analysed post-operatively using FMI to confirm clear margins. Compared to traditional surgical methods, FMI-BCS could be used to precisely visualise and resect orthotopic and metastatic breast tumours. With regulatory approval of these imaging agents, we envision that this robust method could provide an alternative opportunity to improve the detection of the tumour margins and distinguish breast tumours with different molecular classifications in the near future.

## Results

### *In vivo* biodistribution of the fluorescent probes

**Figure 1:**
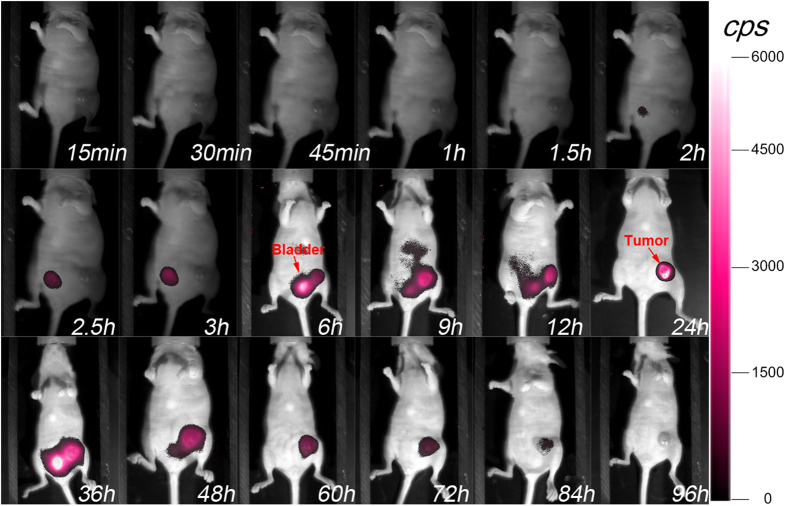
Fluorescence signal distribution of the MMP-750 probe *in vivo* at different time points after intravenous injection of the probe using the Berthold equipment.

In order to determine the feasibility of tumour targeting and the optimal targeting time for precise surgery, mice were administered the MMP-750 probe via tail vein injection for dynamic imaging of the probe. As shown in Fig. 1 and S1, the fluorescent signal was observed at about 6 h post-injection. About 24 h later, the signal at the breast tumour area reached a plateau and remained stable up to 36 h. The signal then gradually dropped to a minimum value 4 days later. These results indicated that the probe could be used to specifically visualise the microenvironment of orthotopic breast tumours and exhibited prolonged retention at the tumour area to guide surgery. The probe was then washed out through the interstitium, and renal clearance was measured. Different concentrations of MMP probes were also analysed in nude mice to monitor the light intensity in the tumour areas (Figure S2). The HER2-645 probe showed similar results (Figure S3), suggesting that the probe could specifically target the HER2 receptor on the membrane of breast cancer cells. The smart MMP-750 probe, which was cleaved by the MMP enzyme, achieved lower background and higher signal-to-background ratio (SBR) than the always-on HER2-645 probe (Fig. 1 and S4), which is more convenient for increased precision of intra-operative FMI-guided BCS.

### Comparison between FMI-guided BCS and traditional surgery in orthotopic tumours

**Figure 2:**
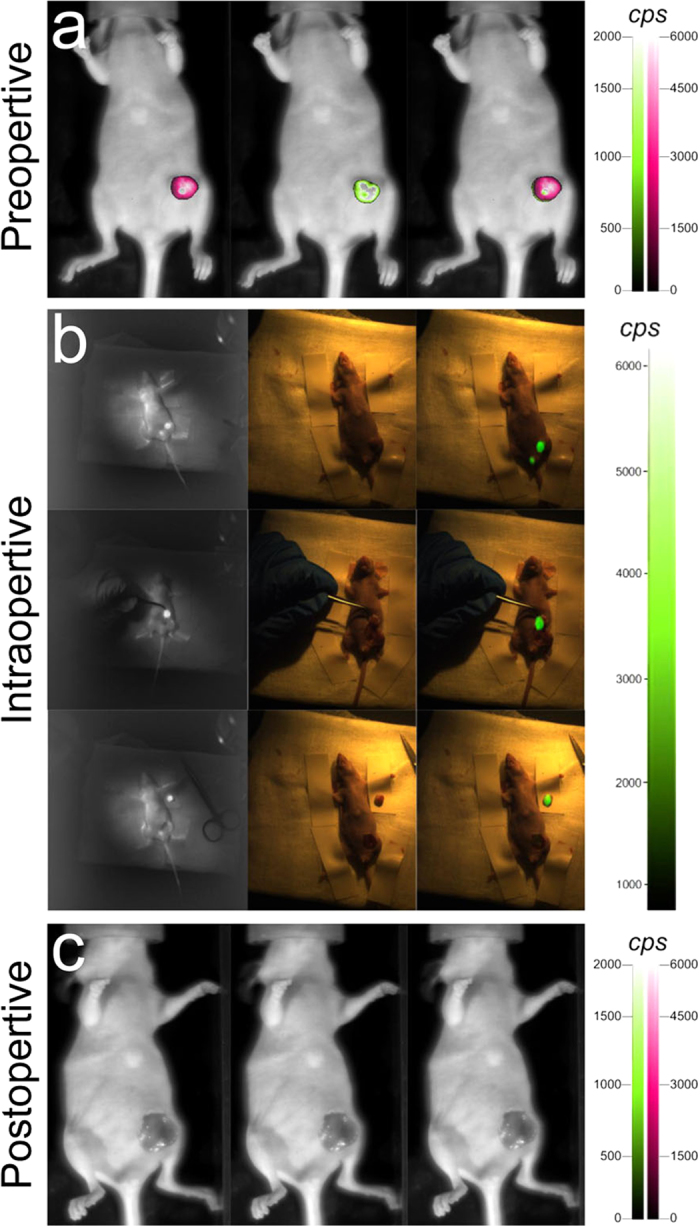
Detection and resection of orthotopic breast tumours using FMI-guided surgery. (**a**) Before surgery, fluorescence and the bioluminescence images were captured to confirm that the probes specifically targeted the breast tumours. The fluorescence signal from the MMP-750 probe was labelled in red. The bioluminescence signal from the 4T1 cell line was labelled in green. Overlays of the probe and bioluminescence signals are shown in the right column. (**b**) The FMI-guided BCS system was used to detect the breast tumour margins and remove the whole tumour intra-operatively. *In vivo* fluorescent images are shown in the first column. Because near-infrared light is not visible, there was no tumour-specific information in colour images *in vivo* in the second column. The merged *in vivo* images based on the calculation of the software in our system are shown in the right column. The lower fluorescent spot is urine, and the light was eliminated by wiping. According to the guidance of the fluorescent image, the surgeons could quickly find the location of the orthotropic breast tumour margin, and the orthotropic tumour could be carefully removed during surgery. (**c**) Fluorescence and bioluminescence images were collected post-operatively to confirm the completeness of the resection.

We next examined whether FMI could delineate clear margins of orthotopic breast tumours *in situ* and further facilitate tumour resection. For early-stage detection of breast cancer (5 days after orthotopic implantation of 4T1-luc breast tumour cells), mice injected with the smart MMP-750 probe underwent FMI-guided surgery, and a separate group of mice underwent traditional surgery. As shown in Fig. 2a, initial post-injection images demonstrated that the fluorescence signal co-registered with the bioluminescence signal, suggesting the ability of FMI to visualise not only the bulk tumour, but also invasive and even irregular tumour margins *in situ*.

Based on the biodistribution results, we chose a time point of 24 h after intravenous injection of the smart probe to perform the surgery using FMI-guided BCS. For mice injected with the MMP-750 probe, surgeons carried out the resection of the orthotopic tumour using our FMI-guided BCS navigation system (Fig. 2b). During the entire operation, the orthotopic breast tumour margin was clearly visualised with our system, assisting the surgeons to perform precise resection of tumours. After surgery, fluorescence and bioluminescence images were captured, and no signal could be observed throughout the entire body of the mouse (Fig. 2c), indicating that the breast cancer tumours were completely removed using visual inspection.

**Figure 3:**
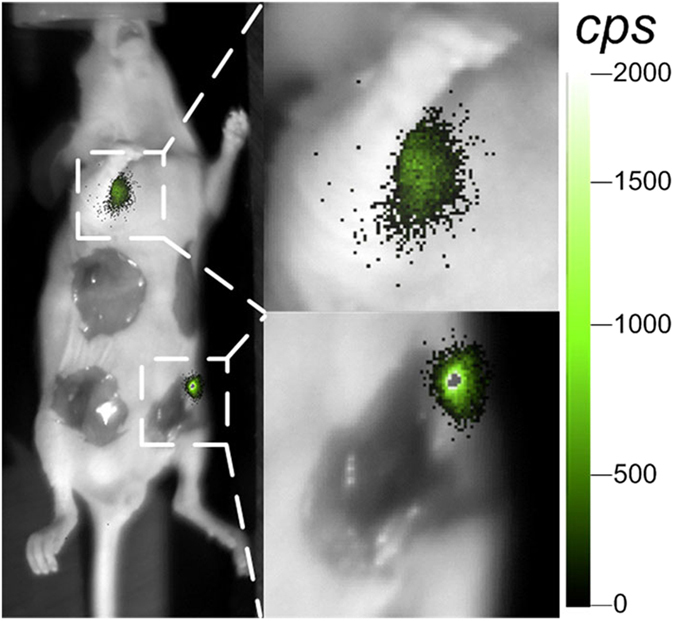
Traditional surgery of mice with the breast tumour. Positive breast tumour tissue and the metastatic tumour were found at the orthotopic tumour margin area and axillary region, respectively, after traditional surgery.

However, for mice undergoing traditional surgery without MMP-750 injection, orthotopic breast tumours were dissected according to the surgeons’ experience and perception. Bioluminescence evaluation was carried out to determine whether the tumour was precisely resected, as shown in Fig. 3. After the surgery, positive tumour tissue was still observed at the orthotopic breast tumour margin area. One small axillary metastatic tumour was found through bioluminescence imaging in the same mouse (Fig. 3); this could not be detected by the naked eye during traditional surgery, even by three experienced surgeons. Moreover, traditional surgery dissected more healthy tissue than FMI-guided surgery. As a result, the FMI method could achieve increased precision of surgery compared to traditional surgery by the same surgeon.

Given that accurately distinguishing the breast cancer subtype intra-operatively could provide immediate information to the surgeon and oncologist regarding further optimal chemotherapeutic options, we used the “always-on” HER2-645 probe, which can specifically target HER2, to detect the subtype of the breast cancer, as shown in Figure S5.

### Intra-operative detection and resection of metastatic tumours using FMI-BCS

**Figure 4:**
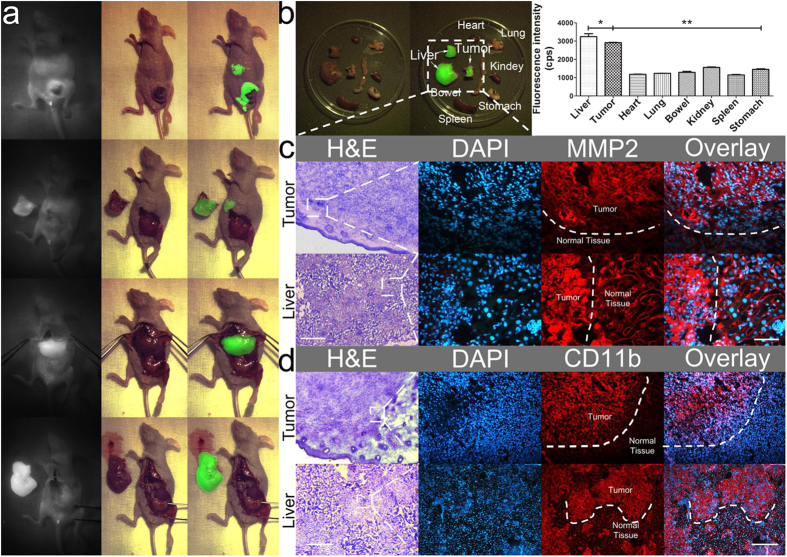
Detection and FMI-guided surgical resection of orthotopic and metastatic 4T1-luc breast tumours in mice. (**a**) Intra-operative images of breast tumour detection *in vivo*. The first two lines are the result of intra-operative dissection of orthotopic tumours, and the lower two lines are resection of liver metastatic tumours during surgery. (**b**) Anatomical results of major organs in mice and quantification of the light intensity in different organs are shown in the right panels. *P < 0.05; **P < 0.001. (**c**) H&E and MMP2 immunofluorescence staining of the orthotopic tumours and liver metastases. H&E: scale bar = 200 μm, 10×; DAPI, MMP2, and Overlay: scale bar = 50 μm, 40×. (**d**) H&E and CD11b immunofluorescence staining of orthotopic tumours and liver metastases. H&E: scale bar = 200 μm, 10×; DAPI, MMP2, and Overlay: scale bar = 50 μm, 40×.

In the clinic, precise location of the margins of metastatic tumours during surgery greatly influences patient prognosis. Herein, we used a metastatic breast cancer model to further evaluate the function and efficacy of FMI-guided BCS system (Table 1). About 25 days after tumour cell implantation, visible orthotopic breast tumours were formed in the lower right breast area of the mice, and metastatic tumours were found in the liver, stomach, and abdomen in different mice under the guidance of bioluminescence imaging. We used a mouse model with orthotopic and liver metastatic tumours to perform sequential dissection with the FMI-BCS system. As shown in Fig. 4a, orthotopic tumours were visualised in the lower right breast area with NIR fluorescence imaging. After removal of the tumour, no fluorescence signal was detected at that area. However, one strong fluorescence signal was found in the abdomen of mice due to the formation of metastatic tumours in the liver. After a quick discussion among surgeons, further surgical removal was carried out. Under fluorescence guidance, all liver metastases were resected. With sequential resection steps, a high congruency between resected tumour tissue and presence of fluorescence signal was noted.

To examine whether the orthotopic and metastatic tumours were completely removed, major organs of the mice were post-operatively dissected, and their fluorescence signals were quantitatively analysed. As shown in Fig. 4b, almost no fluorescence was observed from the other organs except the orthotopic tumour and liver metastasis. Therefore, FMI-guided surgery could help surgeons objectively identify tumour margins and completely dissect orthotopic tumours and additional metastases during surgery.

### Pathological evaluations

To further evaluate the detection of tumour margins *in vivo*, tumour nodules dissected during surgery were analysed with haematoxylin & eosin (H&E) and immunofluorescence staining for MMP2, CD11b (the marker for tumour-associated macrophages), and HER2. As shown in Fig. 4c,d, and S6, tumour margins could be clearly distinguished from H&E staining (indicated with a white dashed line). These results were consistent with the immunohistochemistry results of MMP2, CD11b, and HER2, which were more highly expressed in the tumour sites than in healthy tissues.

### Survival rates following FMI-guided BCS and traditional surgery

**Figure 5:**
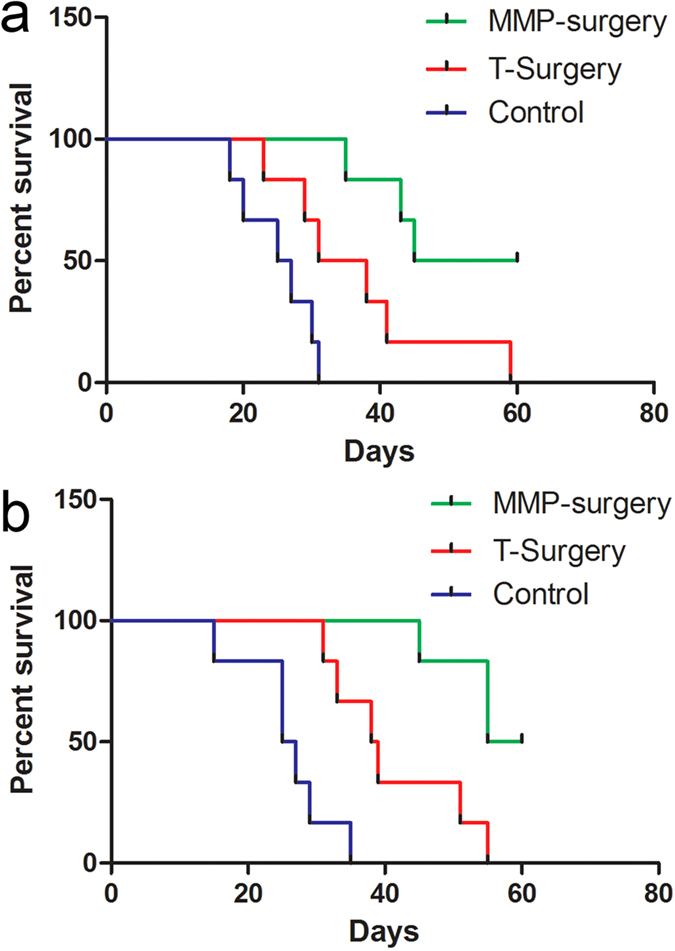
Survival rates of mice from the five groups. (**a**) Survival rates in the orthotopic model. For mice undergoing surgery following MMP-750 injection versus mice undergoing traditional surgery, Wilcoxon test, *P *= 0.03. For the MMP-750 probe versus control method, Wilcoxon test, *P *= 0.0013. (**b**) Survival rates in the metastatic model. For mice injected with the MMP-750 probe versus mice undergoing traditional surgery, Wilcoxon test, *P *= 0.0109. For mice injected with the MMP-750 probe versus the control group, Wilcoxon test, *P *= 0.0013.

Finally, to assess whether FMI-guided surgery could improve the survival of mice, we compared the survival rates of mice (n = 6 in each group) undergoing FMI-guided BCS to that of mice undergoing traditional surgery. As shown in Fig. 5a, the survival rates of mice in the FMI-guided BCS group were significantly higher than the survival rates of mice in the traditional surgery and control groups. Within 60 days, the survival rates in mice undergoing FMI-guided BCS were 50% higher than those in mice undergoing traditional surgery. This improvement may be highly significant in the clinic. With regard to metastasis results using FMI-guided BCS, as shown in Fig. 5b, the survival rate was also increased by 50% (3/6) compared to the traditional surgery method.

### Discussion

The development of more precise medical diagnosis and treatment concepts has revolutionised the treatment of breast cancer. NIR FMI-guided BCS has the potential to improve patient management by visualising breast tumour margins in real time, thereby increasing the completeness of surgery and decreasing the morbidity associated with damage to normal structures. Intra-operative imaging requires a synchronous interplay among contrast agents, tumour biology, imaging systems, and image-analysis algorithms.

In this study, our surgical navigation system was updated to enhance convenience for clinical use. For example, imaging fusion algorithms were improved for real-time image registration^30^. Thus, FMI-guided BCS could be a simple and powerful tool to assist surgeons in the accurate identification of tumour margins and precise intra-operative resection of tumours. Compared to the results of bioluminescence imaging and the gold standard pathological method, our FMI-guided BCS method offered precise detection of the orthotopic tumours and metastases intra-operatively and in real time.

MMPs play an important role in the tumour microenvironment and affect orthotopic and metastatic tumour progression; thus, non-invasive intra-operative imaging of MMP allows for significant improvement of detection of tumours on the (sub)millimetre scale. Furthermore, imaging of the breast cancer cell surface receptor HER2 could provide additional evidence of breast tumour detection and could distinguish the breast cancer subtype for oncologists to carry out additional therapies. At present, the use of fluorescent probes is a major challenge in tumour margin detection for surgical applications. Although the fluorescent dye indocyanine green has been used in clinical practice, it does not have the ability to provide a high signal-to-background ratio due to its nonspecific targeting and “always-on” fluorescence^31^. The preclinical study herein used one smart tumour-specific probe (MMP-750) for intra-operative detection of breast cancer. The MMP-750 probe only responded to the tumour microenvironment but not that of healthy tissues and had low autofluorescence, thus demonstrating lower background and higher contrast than the “always-on” probe. Thus, FMI-guided BCS could provide more information on the characteristics of the specific breast tumour during surgery than current existing methods. As a result, we observed a significant increase in the survival rate of mice that underwent FMI-guided BCS compared to mice that underwent traditional surgery.

For breast cancer patients with liver metastases, if the patient was found to have “primary” liver cancer before surgery, FMI-guided surgery using the HER2 probe will identify the true primary tumour in the breast during surgery and increase the precision of cancer surgery. As the next step, we intend to construct a dual-targeted (MMP and HER2) probe to further improve the sensitivity of tumour margin detection^16^.

In summary, FMI-guided BCS could improve the detection of tumour margins and reduce the presence of residual tumour tissue after surgery, thereby improving prognoses and survival rates. FMI-guided BCS may be a powerful tool for clinical applications in the detection of human breast tumours in the future^32^.

## Methods

### Fluorescence molecular imaging system

FMI-guided surgery was performed using our surgical navigation system, as previously described^33^. Colour video and NIR fluorescence images were simultaneously acquired and dynamically displayed. Based on our previous methods, we further updated our system to provide an improved optical path design, optimised handheld light source, and efficient image fusion algorithm (Table 2). The flexible arm of the collection instrument could be adjusted in any direction above the surgical field during surgery. For enhanced convenient use, the system did not affect the surgical procedure and it was expected to broaden the surgeon’s visual capabilities by providing fluorescence information intra-operatively for a more precise operation.

### Cell culture

4T1-luc cells were cultured in Roswell Park Memorial Institute (RPMI) 1640 (HyClone, Thermo Scientific, USA) supplemented with 10% foetal calf serum (FCS; HyClone, Thermo Scientific) and were maintained at 37 °C in 5% CO_2_.

### Imaging contrast

MMP-750 and HER2-645 imaging contrast probes (PerkinElmer, Inc.) were used for intra-operative imaging. According to the manufacturer’s recommendations, the probes were reconstituted with 1.2 mL of 1× phosphate-buffered saline (PBS) before intravenous injection into animals at a dose of 2 nmol (100 μL) per mouse. The remaining probes were stored at –80 °C and protected from light.

### Orthotopic and metastatic breast cancer mouse models

All animals were purchased from the Department of Experimental Animals, Peking University Health Science Center. All experimental protocols were approved by the Institutional Animal Care and Use Committee (IACUC) at Peking University (Permit Number: 2011-0039), and all the methods were carried out in accordance with the approved guidelines. A total of 1 × 10^6^ 4T1-luc cells in 1× PBS were orthotopically transplanted into the lower right mammary fat pads of 5-week-old athymic female BALB/c nude mice. Tumour growth was monitored based on palpation and bioluminescence imaging (LB983 NC100, Berthold, Germany). As shown in Table 1, mice with tumours growing for 5 days were noted as orthotopic tumour models, while those with tumours growing for 25 days were noted as metastatic tumour models. The mice were anesthetised with an injection of a mixture of ketamine, xylene, and sterile distilled water (0.2 mL) at a ratio of 7:3:4. Based on the biodistribution results, FMI-guided surgery was performed on a small animal operating table (Py2-501213; Harvard, USA) using our surgical navigation system for precise tumour detection after intravenous injection of 100 μL of the MMP-750 probe.

### Biodistribution of the fluorescent probes in mice

In order to evaluate the feasibility of breast tumour detection and the biodistribution time, we randomly chose six nude mice bearing 4T1-luc tumour cells for about 5 days. After injection of the MMP-750 and HER2-645 probes separately, mice were placed in the Berthold equipment for continuous monitoring at different time points.

### FMI-guided surgery

A total of 36 mice were randomly divided into four groups as shown in Table 1. Mice were placed in the Berthold device and monitored for 96 h at the designated detection time after intravenous injection with the MMP-750 probe. For early-stage tumour detection, real-time intra-operative FMI-guided surgery was performed when the orthotropic breast tumour grew sufficiently after cancer cell implantation in the right lower breast area. In order to maintain the fluency of the operation process and reduce the interaction between surgeons and our system, the system started real-time acquisition and automatic recording from the beginning of surgery. Three experienced surgeons removed the orthotopic tumour under NIR fluorescence guidance.

### Pathological evaluation

Breast tumour tissues were excised during surgery and frozen at –80 °C in optimum cutting temperature (OCT) compound (Leica, Germany) immediately after surgery. The tumours were cryosectioned (Leica CM1950, Leica) to yield sections of 4-μm thickness for H&E staining and MMP2 and CD11b immunofluorescence staining. The frozen OCT sections were fixed in acetone for 10 min. Primary mouse anti-human MMP2 antibodies (BD Biosciences, USA) were used to label the tumour environment. Secondary antibody staining was performed using donkey anti-mouse Alexa Fluor 594 antibodies (Invitrogen, Carlsbad, CA, USA). Alternatively, primary rat anti-mouse CD11b antibodies (BD Bioscience) were used to label tumour-associated macrophages in the tumour environment. Secondary antibody staining was carried out using donkey anti-rat Alexa Fluor 594 antibodies. For HER2 evaluation, primary rabbit anti-mouse HER2 antibodies (BD Bioscience) were used to label the orthotopic tumour. Secondary antibody staining was performed using donkey anti-rabbit Alexa Fluor 594 antibodies. The sections were washed twice and mounted with medium containing DAPI (Vector Laboratories, Burlingame, CA, USA).

### Statistical analysis

Data are presented as the average of three independent experiments. One-way analysis of variance (ANOVA) and Tukey’s multiple comparisons tests or Student’s *t*-tests were used to determine significant differences. Differences with *P*-values of less than 0.05 were considered statistically significant. The statistical analysis was conducted using Prism 4.0 (San Diego, CA, USA).

## Additional Information

**How to cite this article**: Chi, C. *et al.* Increased precision of orthotopic and metastatic breast cancer surgery guided by matrix metalloproteinase-activatable near-infrared fluorescence probes. *Sci. Rep.* **5**, 14197; doi: 10.1038/srep14197 (2015).

## Supplementary Material

Supplementary Information

## Figures and Tables

**Table 1:** Experimental groups of mice.

Group	Imaging agent	n	Tumour growthtime (d)	Surgical procedures
MMP-surgery	MMP-750	6	5^a^	Image-guided surgery
		6	25^b^	
T-surgery	None	6	5	Traditional surgery
		6	25	
Control	None	6	−	No surgery

MMP-surgery: FMI-guided breast tumour surgery with the smart MMP-750 probe.

T-surgery: traditional surgery.

^a^Early-stage breast orthotopic tumours.

^b^Orthotopic and metastatic tumours.

**Table 2:** Specifications of the surgical navigation system.

System Features	Performance parameters
Chip area	NIR CCD 1.3′′; Colour CCD 1′′
Lens focal length (mm)	24–120
Working distance (mm)	>600
Surgical field of view (mm)	50 × 50 to 250 × 250
Sensor resolution (spatial)	1024 × 1024 imaging pixels
	13 × 13 μm pixels
	13.3 × 13.3 mm imaging area (optically centred)
Sensor resolution (temporal)	25 fps (full frame)
Image windows	Visible, fluorescent, overlay
