# Supplementary material for: Increased precision of orthotopic and metastatic breast cancer surgery guided by matrix metalloproteinase-activatable near-infrared fluorescence probes
Source: Sci Rep. 2015 Sep 23;5:14197. doi: 10.1038/srep14197 (PMC4585795; doi:10.1038/srep14197)
Supplement: Supplementary Information [file srep14197-s1.pdf]

Supplementary Information

Increased precision of orthotopic and metastatic breast cancer surgery guided by matrix metalloproteinase activatable near-infrared fluorescence probes

*Chongwei Chi[1], Qian Zhang[2], Yamin Mao[1], Deqiang Kou[3], Jingdan Qiu[3], Jinzuo Ye[1], Jiandong Wang[3], Zhongliang Wang[*2], Yang Du[*1], and Jie Tian[*1]*

[1]Key Laboratory of Molecular Imaging of Chinese Academy of Sciences, Institute of Automation, Chinese Academy of Sciences, Beijing 100190, China;

[2]School of Life Science and Technology, Xidian University, Xi'an 710071, China;

[3]Department of General Surgery, General Hospital of People's Liberation Army, Beijing 100853, China.

**Corresponding authors:*

Dr. Zhongliang Wang, Email: wangzl@xidian.edu.cn

Dr. Yang Du, Email: yang.du@ia.ac.cn

Dr. Jie Tian, E-mail: tian@ieee.org; jie.tian@ia.ac.cn

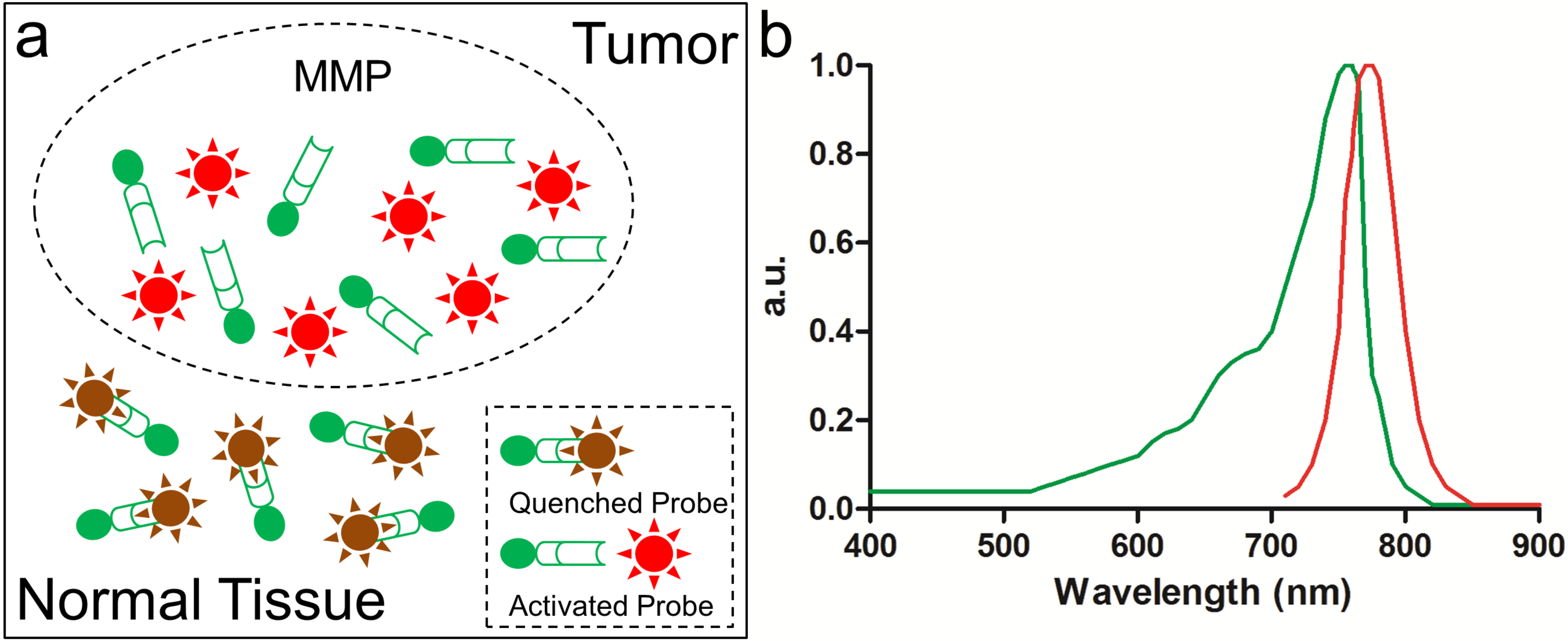

Figure S1. Schematic diagram of the probe targeting property. (a) The tumor area was shown in the dotted line. When the smart MMP-750 probe was injected into the normal tissues, the fluorescence was quenched as shown in brown color. When the smart probe reached to the MMP-overexpressing tumor area, the probe was activated by the cleavage of MMP enzyme and emitted NIR fluorescence. In this case, the excitation and emission spectra of activated MMP-750 probe were shown in the Figure S1b. (λ_{Ex} = 749 nm, λ_{Em} = 775 nm).

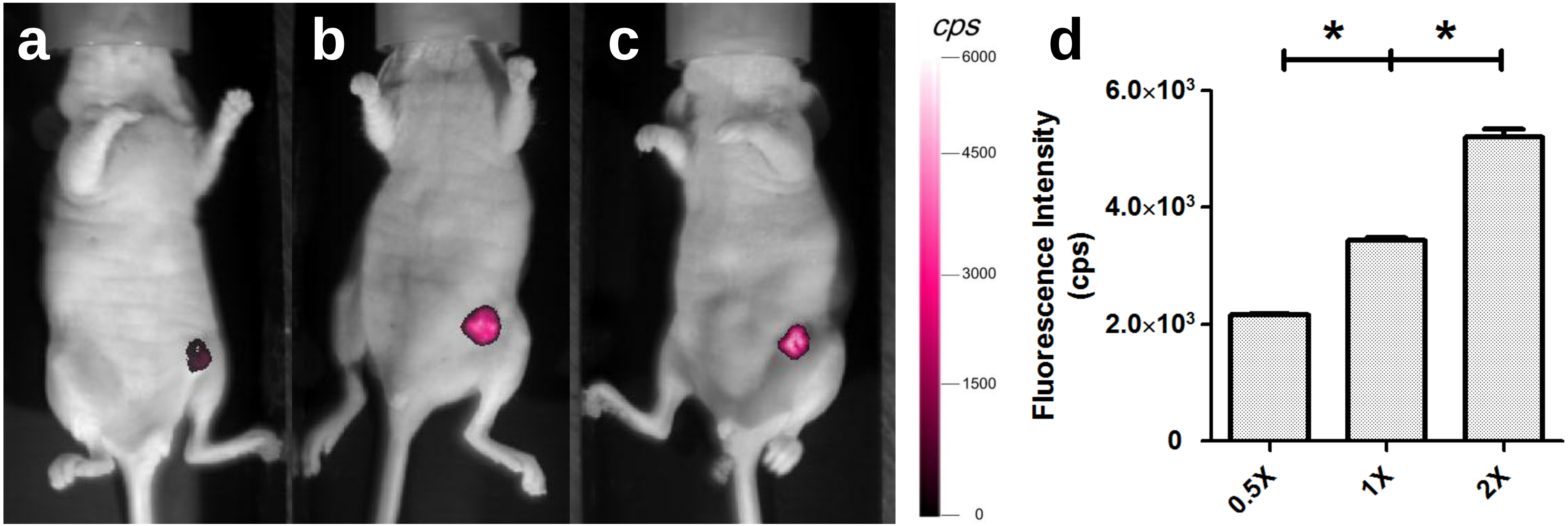

Figure S2. Fluorescence Molecular Imaging of MMP-750 probe24 h after IV injection into the orthotopic breast tumor models. (a) half dosage (1 nmol, 100 µL), (b) recommended dosage (2 nmol, 100 µL), and (c) double dosage (4 nmol, 100 µL). (d) The quantified analysis of the tumor area, which showed statistics significance ($p<0.05$) between different groups. Mean and standard deviation were derived from three independent experiments.

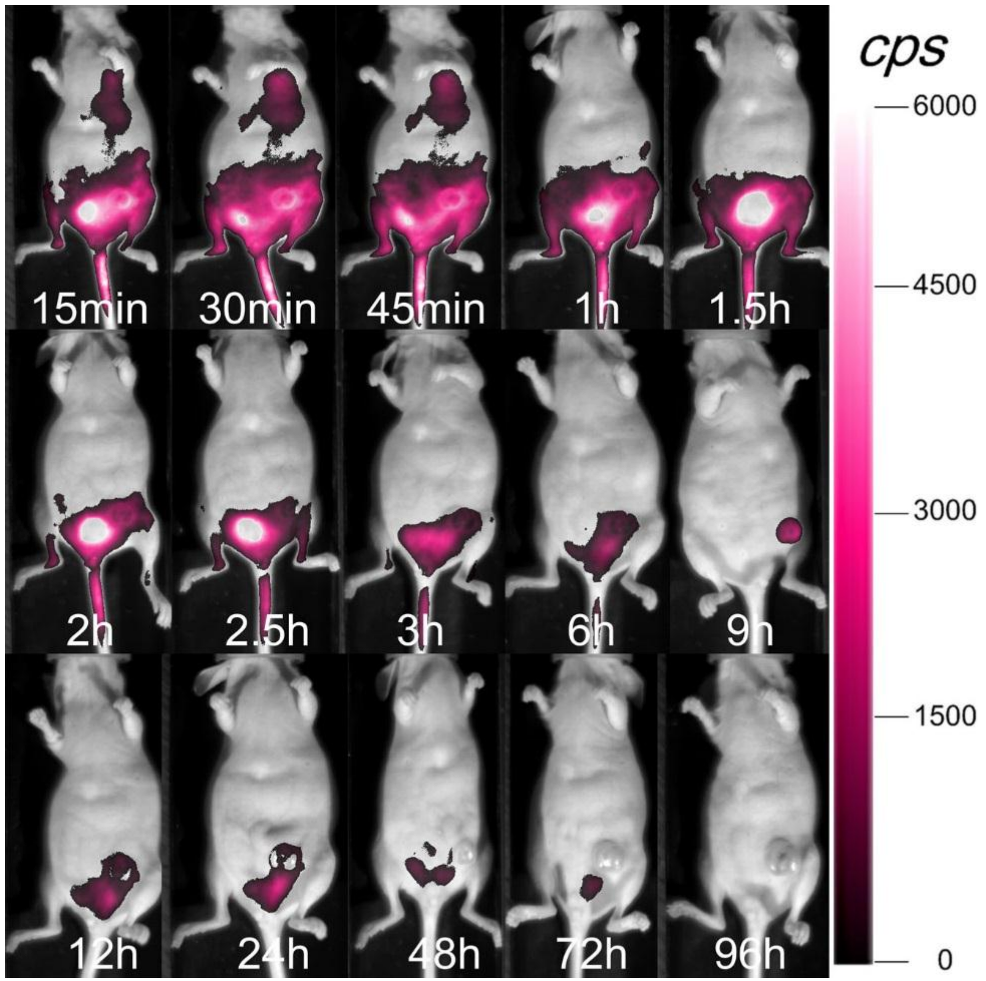

Figure S3. Biodistribution of the HER2-645 probe *in vivo* at different time points after IV injection using the Berthold equipment.

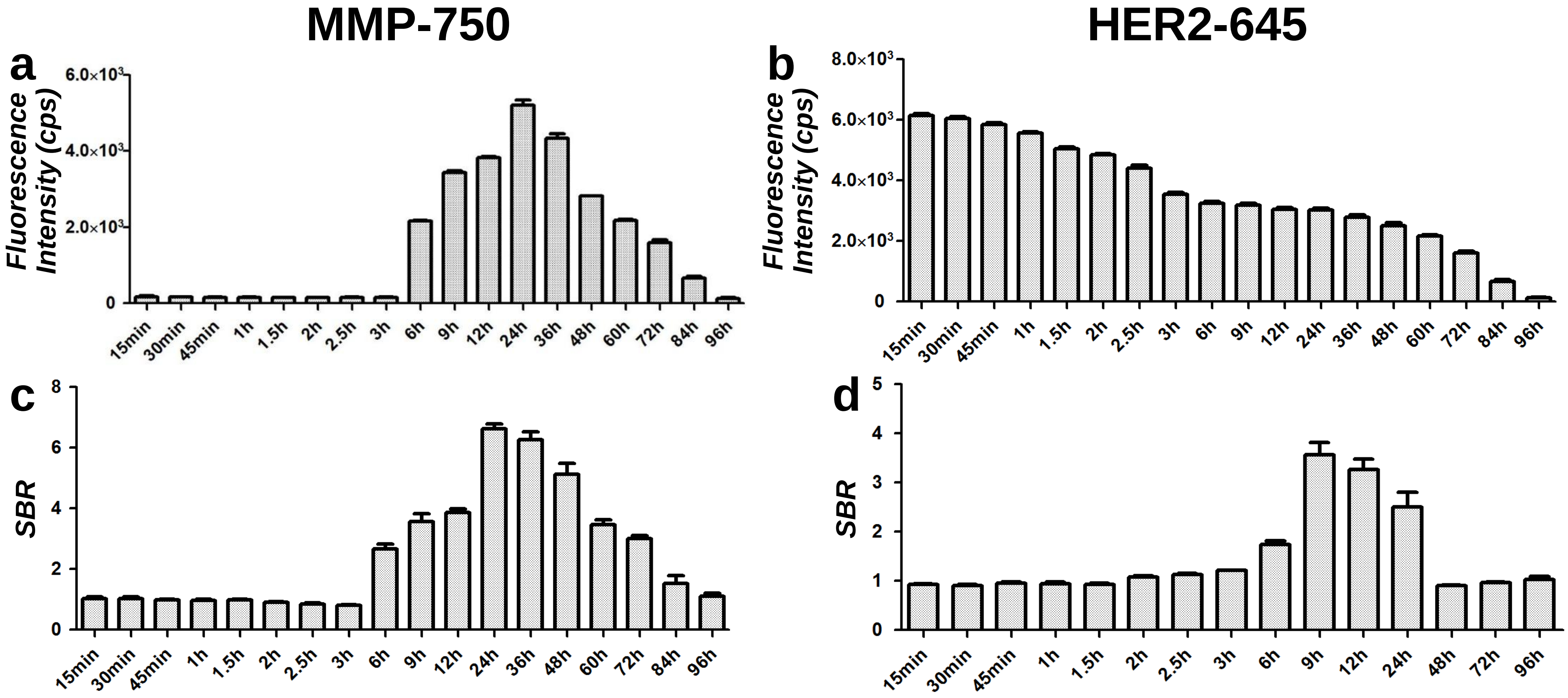

Figure S4. Quantification of the in vivo biodistribution of the MMP-750 and HER2-645 probes at different time points. The quantification of fluorescence intensity in tumor areas with MMP-750 probe (a) and HER2-645 probe (b). Signal to background ratio (SBR) analysis of MMP-750 probe (c) and HER2-645 probe (d).

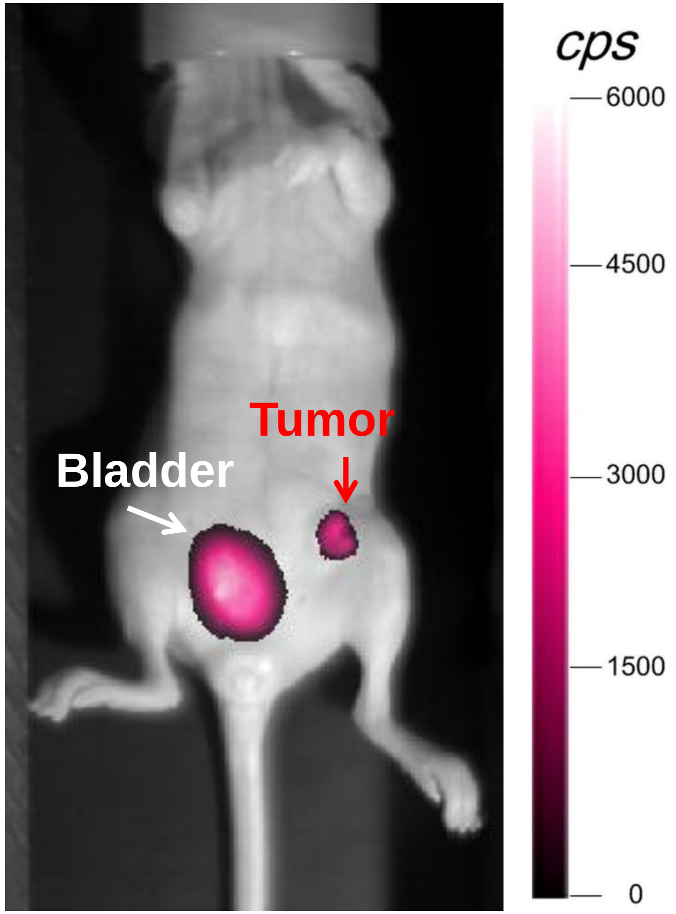

Figure S5. Intra-operative FMI-guided BCS results using HER2-645 probe.

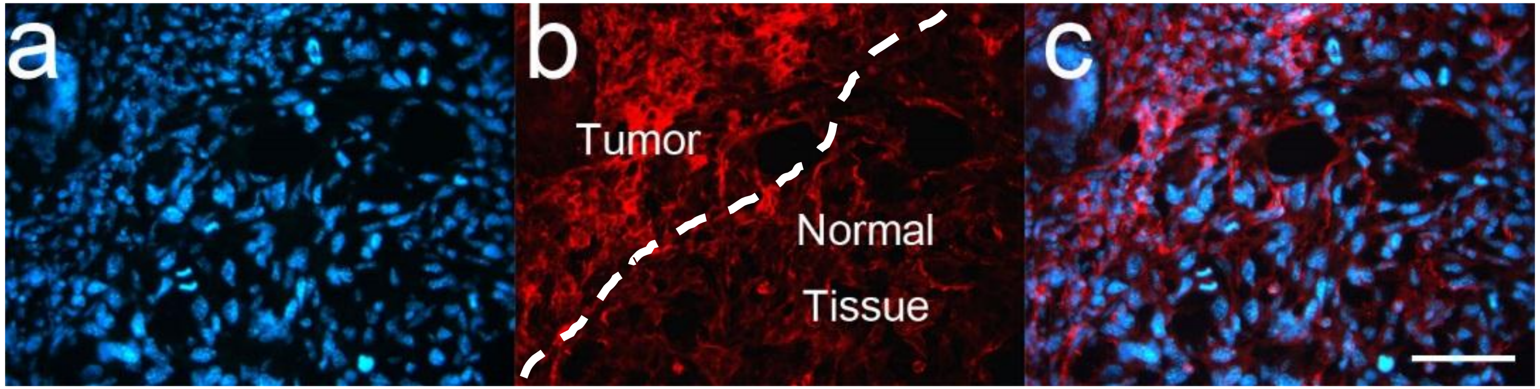

Figure S6. HER2 immunofluorescence staining on orthotopic tumor. (a) DAPI, (b) HER2 immunofluorescence, and (c) overlay. scale bar = 50 μm.
